# Supplementary material for: Trends in antimicrobial resistance amongst Salmonella Paratyphi A isolates in Bangladesh: 1999–2021
Source: PLoS Negl Trop Dis. 2023 Nov 8;17(11):e0011723. doi: 10.1371/journal.pntd.0011723 (PMC10659154; doi:10.1371/journal.pntd.0011723)
Supplement: S1 Table — (DOCX) [file pntd.0011723.s001.docx]

**S1 Table:** Frequency of *Salmonella* Paratyhi A and their corresponding proportions stratified by study sites.

| **Study settings** | **Frequency (%)** |
| --- | --- |
| BSHI | 712 (26.1) |
| SSFH | 214 (7.9) |
| PDC1 | 1634 (60) |
| PDC2 | 87 (3.2) |
| PDC3 | 78 (2.9) |
| Total | 2,725 (100) |
