# Supplementary material for: Trends in antimicrobial resistance amongst Salmonella Paratyphi A isolates in Bangladesh: 1999–2021
Source: PLoS Negl Trop Dis. 2023 Nov 8;17(11):e0011723. doi: 10.1371/journal.pntd.0011723 (PMC10659154; doi:10.1371/journal.pntd.0011723)
Supplement: S2 Table — (DOCX) [file pntd.0011723.s002.docx]

**S2 Table:** Cases of *Salmonella* Paratyphi A and their proportional distribution across different age groups.

| **Age group** | **Frequency (%)** |
| --- | --- |
| Neonates (0 to 1 months) | 3 (0.11) |
| Infants (1 to 11 months) | 57 (2.09) |
| Children (1 to 12 years) | 1,747 (64.11) |
| Adolescents (13 to 17 years) | 143 (5.25) |
| Adults (18 to 65 years) | 676 (24.81) |
| Older adults (≥65 years) | 6 (0.22) |
| No data for age | 93 (3.41) |
| Total | 2,725 (100) |
