# Supplementary material for: Trends in antimicrobial resistance amongst Salmonella Paratyphi A isolates in Bangladesh: 1999–2021
Source: PLoS Negl Trop Dis. 2023 Nov 8;17(11):e0011723. doi: 10.1371/journal.pntd.0011723 (PMC10659154; doi:10.1371/journal.pntd.0011723)
Supplement: Attachment — Submitted filename: Response_to_reviewers_Sajibetal.docx [file pntd.0011723.s004.docx]

Reviewer's Responses to Questions

Key Review Criteria Required for Acceptance?

As you describe the new analyses required for acceptance, please consider the following:

Methods

-Are the objectives of the study clearly articulated with a clear testable hypothesis stated?

-Is the study design appropriate to address the stated objectives?

-Is the population clearly described and appropriate for the hypothesis being tested?

-Is the sample size sufficient to ensure adequate power to address the hypothesis being tested?

-Were correct statistical analysis used to support conclusions?

-Are there concerns about ethical or regulatory requirements being met?

Reviewer #1: The authors set out to investigate the antimicrobial susceptibility of 2725 Salmonella Paratyphoid A isolates collected from multiple sites in Dhaka from 1999 through 2021, making this one of the largest studies on trends and patterns of AMR of Salmonella Paratyphi. The rationale is that the Paratyphoid treatment is commonly based on the available antimicrobial susceptibility data of the more prevalent Salmonella Typhi, which has extensive MDR phenotype. Lack of adequate laboratory infrastructure in endemic countries makes it difficult to sustain routine surveillance for AMR for this organism, so credible AMR data would go a long way to define reliable empiric treatment options.

For some isolates, the team performed both Disk Diffusion and MIC determination for antimicrobial susceptibility, for others, they abstracted historical data from the archives. It is important to explain why for some antimicrobials MICs were performed while for others only disk susceptibility data was available.

**Thank you for providing the constructive review and the suggestion. We have now included an explanation for the undertaking MICs for ciprofloxacin and ceftriaxone (lines 160-163):**

**“MIC determination were conducted for ciprofloxacin and ceftriaxone as most of the isolates in our biobank were resistant to these antibiotics through disc diffusion. MIC determination provides a greater resolution and allows for the detection of subtle changes in susceptibility amongst non-resistant isolates.”**

**MIC determination was also conducted for azithromycin and rationale for this is also included in the revised draft (lines 214-217)**

**“However, previous work has shown that disc diffusion method can detect azithromycin susceptible strains with 100% specificity and 100% sensitivity, but the sensitivity of detection of resistant strains is much lower [28,29]. Therefore, all isolates interpreted as resistant using disc diffusion were retested using MIC strips.”**

Reviewer #2: -Are the objectives of the study clearly articulated with a clear testable hypothesis stated?

Yes

-Is the study design appropriate to address the stated objectives?

Yes

-Is the population clearly described and appropriate for the hypothesis being tested?

Yes

-Is the sample size sufficient to ensure adequate power to address the hypothesis being tested?

Yes

-Were correct statistical analysis used to support conclusions?

Yes, but further methods details are required (as detailed below).

-Are there concerns about ethical or regulatory requirements being met?

No

Minor comments:

- Line 124: PDC3 is mentioned twice but I think one of those mentions should refer to different clinic.

**Thank you for nothing this. This was a mistake and we have now corrected this (line 126).**

- Line 138: Please provide a citation for the biochemical tests or a brief description.

**Thank you. We have added names of the tests routinely performed (Klingler’s Iron agar, Simmon’s citrate agar, motility-indole, and urea agar tests) in out laboratory. (lines 140-141)**

- Line 144: I am not sure if ‘abstracted’ should be ‘extracted’.

**We have revised abstracted to extracted (Line 147)**

- Figure 3: Please also add text to the methods section describing the regression techniques used to generate the black lines.

**Thank you for the comment. We have added a new section (Statistical analysis) (lines 168-172)**

**“R 4.2.3 base functions, ggplot2, dplyr, maps and maptools were used for data analysis and generating figures and maps. To understand possible non-linear trend of ciprofloxacin and ceftriaxone, generalized additive model (mgcv::gam) was utilized with the formula y ~ s(x, bs = “cs”) for >1,000 observations per antibiotic (ciprofloxacin n = 1,205 and ceftriaxone n = 1,208).”**

--------------------

Results

-Does the analysis presented match the analysis plan?

-Are the results clearly and completely presented?

-Are the figures (Tables, Images) of sufficient quality for clarity?

Reviewer #1: No single isolate was resistant to all three first-line drugs at the same time, indicating no circulation of multi-drug resistant (MDR) Salmonella Paratyphi A isolates, but more than 98% of the Salmonella Paratyphi A isolates were non-susceptible to fluoroquinolone. This was likely driven by overtreatment and irrational use of quinolones to treat enteric fever all over South Asia and is often mediated by single point

mutations in quinolone-resistance determining region (QRDR). Good if the team could provide some evidence for this assertion.

**Thank you for your suggestion. In the specified section (lines 243-245), we have included references that emphasize the role of QRDR mutations in DNA gyrase and topoisomerase genes as the primary cause of quinolone non-susceptibility among *S.* typhi and Paratyphi A in South Asia and Bangladesh. These include: “Tanmoy AM, Hooda Y, Sajib MSI, da Silva KE, Iqbal J, Qamar FN, et al. Paratype: a genotyping tool for *Salmonella* Paratyphi A reveals its global genomic diversity. Nat Commun. 2022” and “Rahman SIA, Nguyen TNT, Khanam F, Thomson NR, Dyson ZA, Taylor-Brown A, et al. Genetic diversity of *Salmonella* Paratyphi A isolated from enteric fever patients in Bangladesh from 2008 to 2018. PLoS Negl Trop Dis. 2021”**

**Our attempt to elucidate the potential link between antibiotic resistance resistance and excessive treatment is presented in the subsequent passages (now lines 248-251). -> “The majority (> 90%) of enteric fever patients are treated in the community empirically, and previous work has shown that for each confirmed case of typhoid fever, 3–25 patients without a confirmed detection of typhoidal *Salmonella* are treated with antimicrobials [18]”**

Reviewer #2: -Does the analysis presented match the analysis plan?

Yes

-Are the results clearly and completely presented?

Yes, but the underlying data have not been made available with the manuscript (as detailed below).

**Thank you for suggestion, we have now included one supplementary dataset and two supplementary tables. Supplementary dataset includes metadata of the isolates used in this study for analysis**

**Supplementary table 1: Frequency of Salmonella Paratyphi A and their corresponding proportions stratified by study sites.**

**Supplementary table 2: Cases of Salmonella Paratyphi A and their proportional distribution across different age groups.**

-Are the figures (Tables, Images) of sufficient quality for clarity?

Yes, however some modifications wold aid clarity (as detailed below).

**Thanks for suggestion, we have revised the figures according to suggestions below.**

Minor comments:

- Line 183: Please briefly define MIC50 & MIC90.

**Thank you. We have provided definitions of MIC50 & MIC90 (Now line 201-203)**

**-> “MIC50 (concentration that inhibits ≥50% of the isolates) was 0.5 μg/mL and MIC90 (concentration that inhibits ≥90% of the isolates) was also 0.5 μg/mL.”**

- Lines 208-210: Please consider providing a supplementary figure that stratifies the data by clinic to help illustrate the importance of this sampling. Similarly, please also consider stratifying the data by adult and child cohorts if the data are available to do so.

**Thank you for the suggestion. We have made two tables for this (can be found in the Supplementary_document table 1 and 2)**

- Figure 2: Please provide the denominator values for the samples examined each year (for the total of n=2725). This could potentially be done using a second y-axis and a line graph. Please also consider including the untested sequences as another colour in this plot to show the frequencies of missing AST data.

**Thank you for noting this. We have revised the figure accordingly and have updated figure 2 by adding another colour to show the proportion of untested samples**.

- Figure 3: Please define the blue lines & points in the figure legend.

**Thank you for the suggestion. We have revised the figure legend.**

**->Each individual dot corresponds to a single isolate and its colour highlights the interpretation of phenotypic resistance as per CLSI-2022 guidelines.**

**~~Note, we noticed a small typo in this figure legend. GAM was used to generate the black line. Corrected accordingly. Please check Methods for details (Line 167-169).~~**

- Please make a spreadsheet of the data available as a supplementary file.

**We have now created a supplementary data file (Supplementary_dataset)**

--------------------

Conclusions

-Are the conclusions supported by the data presented

-Are the limitations of analysis clearly described?

-Do the authors discuss how these data can be helpful to advance our understanding of the topic under study?

-Is public health relevance addressed?

Reviewer #1: The major conclusion is the fact that S. Paratyphi A isolates have a completely different pattern of AMR compared to S. Typhi, the former being mostly pan-susceptible to all commonly available antimicrobials and therefore these drugs could be used for empiric treatment of paratyphoid. But in addition, the high fluoroquinolone non-susceptible proportion of isolates clearly shows concern for across serovar transmission of resistance genes.

Reviewer #2: -Are the conclusions supported by the data presented?

Yes

-Are the limitations of analysis clearly described?

Yes

-Do the authors discuss how these data can be helpful to advance our understanding of the topic under study?

Yes

-Is public health relevance addressed?

Yes

--------------------

Editorial and Data Presentation Modifications?

Use  this section for editorial suggestions as well as relatively minor modifications of existing data that would enhance clarity. If the only modifications needed are minor and/or editorial, you may wish to recommend “Minor Revision” or “Accept”.

Reviewer #1: N/A

Reviewer #2: - Line 57: ‘Year’ should be ‘years’.

**Revised.**

- Line 85: It would give further context to the work if the authors could briefly state the current stage of vaccine development for S. Paratyphi A or refer to a recent review on the topic.

**Thank you for this suggestion. We have updated this section accordingly and added the following reference -> “Shakya M, Neuzil KM, Pollard AJ. Prospects of Future Typhoid and Paratyphoid Vaccines in Endemic Countries. J Infect Dis. 2021”. (lines: 86-87)**

- Line 86/107: Please provide a citation for the statement regarding declining typhoid cases.

**Line 86: Please refer to Dong B-Q et al, 2010. (Reference included lines: 87-90 now)**

**Line 107: Here we assumed that the cases are likely to reduce in the post TCV-era as this vaccine has proven to be highly efficacious. (Changed “as” to “if” for clarification clarify: Now Line: 108)**

- Line 171: Please consider rephrasing this to refer to Dhaka rather than all of Bangladesh, unless suitable data from other regions support the notion of extrapolating these findings to Bangladesh more broadly.

**Revised accordingly. (Now line 190)**

- Line 224: ‘Majority’ should be ‘The majority’. Please also provide a citation for the lack of blood culture infrastructure in LMIC settings.

**Thank you, revised to “The majority” (Now line 248)**

**We have added two references for this. -> Saha SK, Tabassum N, Saha S. Typhoid Conjugate Vaccine: An Urgent Tool to Combat Typhoid and Tackle Antimicrobial Resistance. J Infect Dis. 2021. doi:10.1093/infdis/jiab443 PMID: 34528685**

**And**

**Saha S, Islam M, Uddin MJ, Saha S, Das RC, Baqui AH, et al. Integration of enteric fever surveillance into the WHO-coordinated Invasive Bacterial-Vaccine Preventable Diseases (IB-VPD) platform: A low cost approach to track an increasingly important disease. PLoS Negl Trop Dis. 2017. doi:10.1371/journal.pntd.0005999 PMID: 29073137**

- Line 236: ‘acrb’ should be ‘acrB’.

**Thank you, revised acrb to acrB (Now line 261)**

- Line 235: ‘Resistance’ should be ‘resistant’.

**Resistance has been revised to resistant (line 259 now)**

- Line 240: It is unclear from the text at present how TCVs contribute to this. I think the intention is that when S. Typhi is adequately controlled with vaccines, empirical treatment for enteric fever could be based on Paratyphi A resistance patterns, but I am not sure. Please consider rephrasing this text to improve clarity.

**Rephrased this part (lines 263-271)**

**“Taken together these findings suggest that empirical treatment of paratyphoid fever with ceftriaxone and azithromycin will continue to be effective. However, our findings also suggest that a successful implementation of TCV could facilitate a shift in the approach to enteric fever treatment. If Salmonella Typhi becomes less prevalent during post-TCV era, and if there is increased availability of rapid diagnostics to detect paratyphoid fever [48] there is potential to base empirical treatment on the susceptibility patterns of Salmonella Paratyphi A. Since none of the Salmonella Paratyphi A in Dhaka exhibited multidrug resistance, first line of drugs like ampicillin, cotrimoxazole, or chloramphenicol can be used empirically for paratyphoid feverwithout affecting the treatment efficacy.”**

--------------------

Summary and General Comments

Use  this section to provide overall comments, discuss strengths/weaknesses of the study, novelty, significance, general execution and scholarship. You may also include additional comments for the author, including concerns about dual publication, research ethics, or publication ethics. If requesting major revision, please articulate the new experiments that are needed.

Reviewer #1: For the Paratyphoid cases from community and hospital setting,the improvement in rapid diagnostics to detect paratyphoid fever could allow for shifting of the empirical treatment of paratyphoid fever to the first line of drugs – ampicillin, cotrimoxazole, or chloramphenicol without affecting the efficacy of treatment. This would allow for preservation of reserve antibiotics including ceftriaxone and azithromycin tfor treatment of other severe infections.

Reviewer #2: Sajib and colleagues report on the trends in AMR from n=2,725 Salmonella Paratyphi A isolates collected over 23 years (1999-2021) from multiple clinics in Dhaka, Bangladesh. This manuscript fills a key knowledge gap regarding the burden of AMR Paratyphi A in Bangladesh, for which historical data are scant. A key finding is that despite popular older examples in the literature, Multidrug resistant S. Paratyphi A was not identified within the study period. The manuscript is well written, analyses appropriate, and the data are important for guiding empirical treatment of paratyphoid fever. The study is also timely given the rollout of typhoid conjugate vaccines in many endemic settings, which, as the authors note, may lead to an increase of paratyphoid cases. However, the manuscript could benefit from some minor edits to improve clarity, and the underlying data should be made available, as outlined in the other sections of this review.
